# Inaugural year of regulated psilocybin services in Oregon: safety, motivations, and utilization

**DOI:** 10.3389/fpsyt.2026.1777387

**Published:** 2026-05-13

**Authors:** Feliciano Yu, Joe Tafur, Francisco Moreno, Stephen Dahmer

**Affiliations:** 1 Department of Pediatrics, University of Arkansas for Medical Sciences, Little Rock, AR, United States; 2 Andrew Weil Center for Integrative Medicine, University of Arizona, Tucson, AZ, United States; 3 College of Medicine, University of Arizona, Tucson, AZ, United States

**Keywords:** drug safety outcomes, patient demographics, psilocybin services, public health surveillance, regulated psychedelic access

## Abstract

**Importance:**

The Oregon Psilocybin Services (OPS) program is the first statewide, regulated framework for legal psilocybin in the U.S. Analyzing inaugural-year utilization and safety is essential for informing policy and equity monitoring.

**Methods:**

We conducted a descriptive analysis of statewide aggregate data from the OPS Public Dashboard (January 1–December 31, 2025). Outcomes included service volume, client demographics, motivations, and acute adverse events.

**Results:**

In 2025, 5,935 clients participated in 5,375 sessions. Volume peaked in Q2 (n=1,758) before stabilizing in Q4 (n=1,358). Service tourism was significant, with 32.6% of participants residing outside Oregon. The largest segment was aged 35–49 (~40%); women (57.4%) and LGBTQ+ individuals (27.2%) represented substantial portions of the annual cohort. Racial diversity was limited, with White participants representing 84.1%–91.5% quarterly, while Hispanic/Latino (7.1%) and African American (2.1%) participation lagged. Adverse events were rare, with annual behavioral and medical rates of 2.42 and 2.79 per 1,000 sessions, respectively.

**Discussion:**

Full-year data indicate stabilized utilization by a predominantly midlife adult population. While the program successfully reaches sexual and gender minorities, racial disparities persist. High service tourism suggests significant socioeconomic barriers. These findings underscore the program’s dual role as a wellness modality and a functional alternative for addressing mental health distress.

## 1 Introduction

The Oregon Psilocybin Services Act (Oregon Revised Statutes 475A), approved by voters as Measure 109 in November 2020, established the first state-regulated framework for psilocybin services in the United States (1, 5). This legislation legalizes supervised psilocybin administration for adults at licensed centers statewide, coinciding with growing interest in naturalistic and clinical trial exploration of psilocybin’s wholistic benefits and its therapeutic potential for conditions such as treatment-resistant depression, substance use disorders, and end-of-life anxiety (1–4).

The Oregon Health Authority (OHA) oversees the Oregon Psilocybin Services (OPS) section, which licenses manufacturers, laboratories, facilitators, and service centers (Oregon Health Authority, 2024). Unlike clinical trials, the act does not require a medical diagnosis for access but mandates administration in controlled settings to maximize safety (1, 5). The OPS framework utilizes a non-medical “facilitation” model consisting of three mandatory components: a preparation session for screening and intention setting, a supervised administration session at a licensed center, and an optional integration session to discuss the experience. This differs from most clinical trials by allowing for varied dosing and the presence of licensed facilitators who may not be medical or mental health professionals.

To ensure monitoring and inform policy, the Oregon Legislature passed Senate Bill 303 (SB 303), codified in ORS 475A.372 and 475A.374 (6). This requires licensed centers to collect standardized, de-identified client data, including REALD (Race, Ethnicity, Language, Disability) and SOGI (Sexual Orientation and Gender Identity) variables, to support equity and outcome monitoring (7, 8).

Despite growing scientific interest, little is known about the demographics and motivations of individuals seeking psilocybin services within a non-medical, state-regulated framework. Furthermore, while early reports focused on initial program rollout, there is a need for a comprehensive assessment of utilization patterns over a sustained period. This study aims to characterize statewide utilization and client demographics during the first full year of regulated psilocybin services in Oregon, examine self-reported motivations with an emphasis on wellness versus mental health use, and estimate rates of acute behavioral and medical adverse events to inform safety monitoring in non-medical psychedelic care models. To date, these publicly available data have not been synthesized to provide a comprehensive annual profile of service users, their reasons for use, and the associated acute safety profile, information that is critical to ongoing national policy discussions regarding regulated psychedelic access (9, 10).

## 2 Materials and methods

### 2.1 Study design and data sources

This descriptive analysis utilized publicly available, aggregate-level data from the OPS Data Dashboard Archives for full 2025 calendar year (Q1: January 1 – Q4: December 31) (13, 37). Data sources included quarterly OPS dashboard CSV files, the 303 Client Data Form, and OPS fact sheets (11, 12). The University of Arkansas for Medical Sciences Institutional Review Board determined this study to be Not Human Subjects Research (IRB Number: 299603) as it utilized only publicly available, aggregate, de-identified data.

### 2.2 Data collection and measures

Service centers are required to submit aggregate totals quarterly via a secure OPS portal. The dataset included 386 variables covering service volume, denials (potential clients who did not meet eligibility and participation parameters), adverse events, and client demographics (11).

The data are de-identified and aggregated at the center level. Consequently, the dataset does not allow for the tracking of individual client trajectories across quarters or the identification of repeated participation (e.g., multiple-dose recipients). All counts for ‘clients served’ represent the sum of individuals reported by centers within that specific reporting period.

#### 2.2.1 Safety events

OPS classifies safety events into four categories: adverse behavioral, severe behavioral, adverse medical, and severe medical reactions. ^12^ An “adverse reaction” is defined as a response requiring emergency services or medical provider contact during a session, while a “severe adverse reaction” requires hospital transport (12, 13).

#### 2.2.2 Demographics

**Figure 1:**
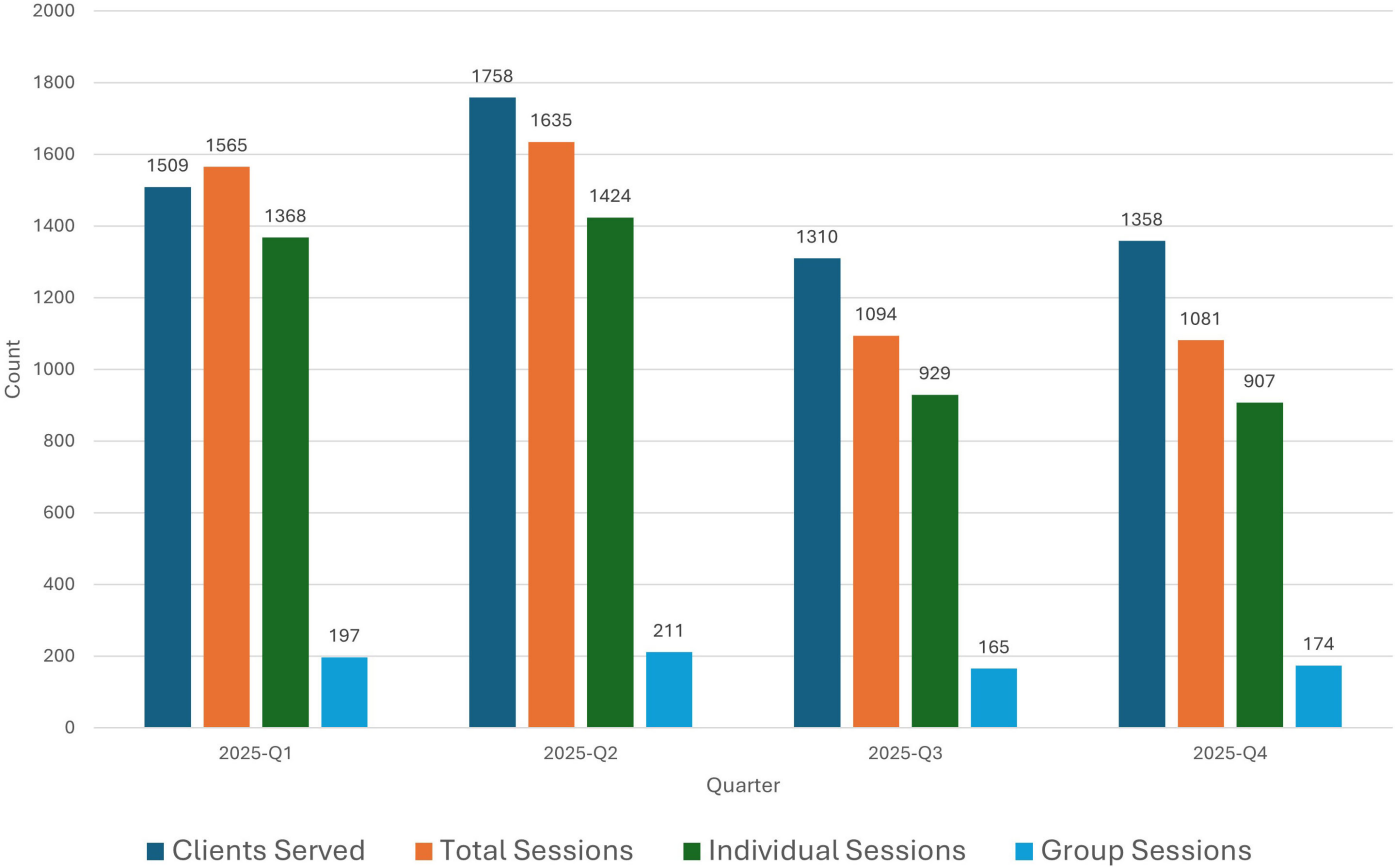
Quarterly service volume for Oregon psilocybin services (Q1–Q4 2025). This figure presents the utilization trends during the inaugural year of regulated psilocybin services. Bars represent the absolute number of clients served, total administration sessions, and the breakdown between individual and group session formats. Data indicate a peak in utilization during the second quarter (Q2) followed by stabilization in the latter half of the year.

Variables included age, gender identity, sexual orientation, and race/ethnicity. To identify peak engagement periods, age data were primary categorized into broader lifecycle cohorts (21–34, 35–49, 50–64, and 65+ years); however, more granular 5-year intervals were maintained for visual trend analysis in **Figure 1**.

#### 2.2.3 Residency and tourism

Client residency was categorized as ‘Oregon,’ ‘Other Inside US,’ or ‘Outside US’ to identify the proportion of service tourism. Detailed annual and quarterly residency metrics are provided in **Supplementary Table 1**.

#### 2.2.4 Reasons for use

Clients utilized a multi-select checklist to indicate reasons for requesting services, including wellness, mental health diagnoses, trauma, and spiritual growth (11). The complete quarterly breakdown of participant motivations is available in **Supplementary Table 2**.

### 2.3 Statistical analysis

All analyses were performed on statewide aggregates. Records with suppressed values (–99), used for confidentiality protection in small cell sizes, were excluded from percentage calculations; therefore, resulting counts and percentages represent minimum estimates (9, 10). Visit reason proportions were calculated by dividing category counts by the total number of clients served per quarter. To improve the precision of safety monitoring, 95% Confidence Intervals (CI) were calculated for behavioral and medical adverse event rates per 1,000 sessions using the Wilson score method to account for small event counts.

## 3 Results

### 3.1 Service volume and safety

During the 2025 calendar year, 5,935 clients were served across 5,375 administration sessions including 747 group administrations with two or more psilocybin recipients per session. Service volume peaked in Q2 (1,758 clients) before stabilizing in the second half of the year (Q3: 1,310; Q4: 1,358). Correspondingly, total administration sessions followed a similar trend, peaking at 1,635 in Q2 (**Figure 1**) (9, 10).

Service denial rates showed a marked decrease over the year, from 8.0% (n = 121) in Q1 to a low of 1.3% (n = 17) in Q3, before rising slightly to 4.3% (n = 58) in Q4. The primary reasons for denial consistently involved client ineligibility or service requests that were inconsistent with a center’s specific business model. Notably, acute intoxication at the time of service was not reported as a cause for denial in any quarter. However, because the aggregate data do not further categorize ‘ineligibility,’ it remains unclear if these denials were related to specific clinical contraindications, such as concurrent medication use or underlying psychiatric conditions. Within the limits of aggregate reporting, adverse events remained rare throughout the annual period (**Table 1**). Within the limits of aggregate reporting, adverse events remained rare throughout the annual period (**Table 1**). The annual behavioral adverse event rate was 2.42 per 1,000 sessions (n = 13), with quarterly rates ranging from a low of 0.93 in Q4 to a peak of 4.57 in Q3. Similarly, the annual medical adverse event rate was 2.79 per 1,000 sessions (n = 15), ranging from 1.85 in Q4 to 5.48 in Q3. Severe reactions requiring hospital transport were minimal; only five severe behavioral and two severe medical reactions were reported across all four quarters (n = 7 total), representing an exceptionally low incidence within the statewide regulated framework.

**Table 1:** Service volume, service denials, and safety outcomes in Oregon psilocybin services, January 1 to December 31, 2025.

Measure	2025 Q1	2025 Q2	2025 Q3	2025 Q4	Annual total
Service volume					
Clients served	1,509	1,758	1,310	1,358	5,935
Individual administration sessions	1,368	1,424	929	907	4,628
Group administration sessions	197	211	165	174	747
**Total administration sessions**	**1,565**	**1,635**	**1,094**	**1,081**	**5,375**
**Average psilocybin dose (mg)** ^a^	**24.44**	Suppressed ^b^	**24.10**	**24.86**	**24.47** ^c^
Service denials					
Denials of psilocybin services (Total)	121	62	17	58	258
*Denial Rate (%)*	8.0%	3.5%	1.3%	4.3%	4.2% ^d^
**Safety Outcomes (Total Events)**					
Adverse behavioral reactions	0	3	2	1	6
Severe behavioral reactions	2	2	3	0	5
Adverse medical reactions	3	3	6	1	13
Severe medical reactions	0	1	0	1	2
Safety event rates (per 1,000 sessions)					
**Behavioral adverse event rate**	**1.28**	**3.06**	**4.57**	**0.93**	**2.42** ^d^
*95% CI (Wilson Score)*	*(0.15–4.62)*	*(0.99–7.14)*	*(1.48–10.67)*	*(0.02–5.15)*	
**Medical adverse event rate**	**1.92**	**2.45**	**5.48**	**1.85**	**2.79** ^d^
*95% CI (Wilson Score)*	*(0.40–5.60)*	*(0.67–6.26)*	*(2.01–11.94)*	*(0.22–6.68)*	

^a^ Dose represents the quarterly mean of all administered sessions; session-level variance (SD) and ranges were not available in the statewide aggregate data.

^b^ Q2 dosing data were suppressed in the source archive due to reporting inconsistencies or privacy thresholds.

^c^ Annual average dose is calculated as the mean of the three available quarterly averages.

^d^ Annual average.

Values in bold represent the primary analysis cohorts and annual summary totals for the 2025 reporting period.

### 3.2 Client demographics

**Figure 2:**
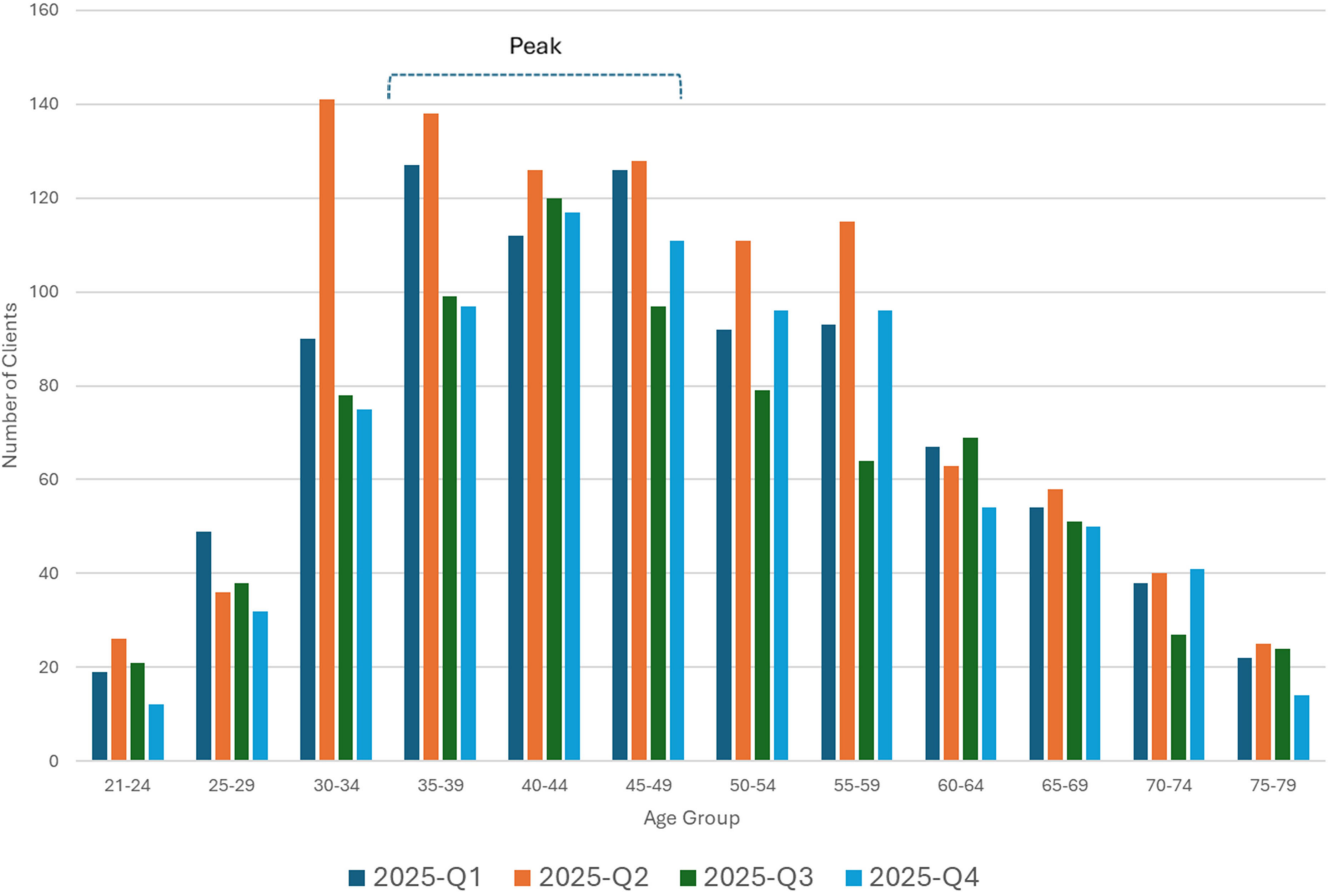
Client age distribution by quarter (Q1 to Q4 2025). This histogram displays participant age distribution in 5-year intervals across the four quarters of the study period. The horizontal bracket identifies the primary analysis cohort (ages 30–49), which consistently represented the peak participation segment. Data highlight a midlife adult majority among early adopters of the regulated psilocybin model.

The 35–49 age range consistently represented the largest client segment throughout 2025, accounting for approximately 40% of all clients served (**Figure 2**). When analyzed by 5-year cohorts, peak engagement across most age groups occurred in Q2, particularly within the 30–34 (n = 141) and 35–39 (n = 138) brackets. Beyond this peak, participation declined significantly with age, as clients 65 and older collectively represented less than 15% of the annual total.

Gender identity reporting improved significantly by the end of the year, with “Missing/No Answer” responses dropping from a high of 175 in Q2 to just 22 in Q4. Women consistently represented the majority of participants (54.5%–59.0%). Sexual orientation data showed that LGBTQ+ representation was highest in Q1 (32.4%) and stabilized between 23.5% and 26.9% for the remainder of the year.

The program demonstrated limited racial and ethnic diversity. Combined White and European subgroups (Western European, Eastern European, and Other White) represented 84.1% to 91.5% of the reporting population across all quarters. Hispanic/Latino representation declined from 9.8% in Q1 to 5.6% in Q4, while African American participation remained low, ranging from 1.9% to 2.4% (**Table 2**).

**Table 2:** Demographic characteristics of clients accessing Oregon psilocybin services (OPS), by quarter (2025 Q1–Q4).

Demographic category	Q1 (n)	Q2 (n)	Q3 (n)	Q4 (n)	Annual total (n)	Annual %
Woman/Girl	522	542	460	477	**2,001**	**57.4%**
Man/Boy	359	395	302	326	**1,382**	**39.6%**
Non-Binary	33	57	18	37	**145**	**3.0%**
Straight	608	741	578	584	**2,511**	**72.8%**
LGBTQ+ Total	292	227	205	215	**939**	**27.2%**
Western Euro	423	431	420	400	**1,674**	**48.6%**
Other White	205	276	178	161	**820**	**23.8%**
Eastern Euro	136	129	123	151	**539**	**15.6%**
Hispanic	89	63	49	44	**245**	**7.1%**
Asian	32	51	0	12	**95**	**2.8%**
African Amer.	22	22	15	15	**74**	**2.1%**

Percentages are calculated using valid responses for each domain (gender identity, sexual orientation, race/ethnicity); missing and suppressed cells (coded as −99) are excluded. Hispanic/Latino combined includes Central American, Mexican, South American, and Other Hispanic categories. Race/ethnicity percentages for the displayed rows use the sum of displayed categories as the denominator (not the full REALD race total). Non-binary+ includes Agender, Bigender, fluid, etc. as defined in your aggregate code.). Values in bold represent the primary analysis cohorts and annual summary totals for the 2025 reporting period.

### 3.3 Reasons for requesting services

Motivations for seeking services remained diverse and stable throughout the year (**Supplementary Table 1**). The most frequently cited reasons included general health and wellness (30.6% annually; quarterly range: 25.4%–35.8%), change of perspective (27.7% annually; 26.1%–30.1%), and expanded consciousness (27.0% annually; 24.3%–29.6%). Mental health concerns were also prominent and stable; anxiety was reported by 23.8% of clients annually (22.5%–25.0%), depression by 22.0% (20.8%–23.7%), and PTSD by 13.1% (11.0%–14.1%).

### 3.4 Service tourism

Analysis of client residency reveals a significant “service tourism” component within the OPS program, with 32.6% of the total annual client base (n = 1,936) originating from outside of Oregon (**Supplementary Table 2**). Domestic travelers from other U.S. states represented the vast majority of this cohort (29.5%, n = 1,753), while international visitors accounted for approximately 3% (n = 183) of the total population. Although the total number of clients served was highest in Q2, the proportion of out-of-state participants peaked in Q3 at 38.5% (504 of 1,310 clients). International participation showed a notable spike in Q2 with 140 clients, though it remained relatively limited and stable in the subsequent quarters.

The average psilocybin dose remained consistent throughout the year, with quarterly averages ranging from 24.10 mg to 24.86 mg (**Table 1**). However, session-level variance and specific dose ranges were not available in the statewide aggregate data.

## 4 Discussion

Full-year data from the OPS program reveal a stabilized pattern of service delivery, with over 5,300 administration sessions completed in 2025. Although this report contains no data on benefit regardless of reason for psilocybin use, the low rate of serious adverse events, specifically the rarity of events requiring hospital transport during the first year of the program, suggests that supervised psilocybin administration may be implemented safely to carefully selected participants who meet medical and psychiatric inclusion criteria, in a regulated, non-medical context. This should be interpreted with caution given that the reports of safety are limited to acutely observable events, the distinction between “behavioral” and “medical” adverse events in aggregate reporting remains somewhat imprecise, and the low event volume should be interpreted with caution regarding the quality and completeness of reporting in a non-clinical framework (14, 15).

Adverse events, whether behavioral or medical, were rare. This aligns with OPS safety communications and clinical trial data, where serious adverse events are uncommon and most reactions are mild and self-limited (7, 16, 17). The low frequency of severe events requiring hospital transport highlights the potential safety of supervised psilocybin consumption within regulated environments, though caution remains essential in interpreting aggregate-level data. While service denial rates decreased between Q1 and Q2, the drivers of this trend, whether related to evolving intake procedures, shifts in client-center alignment, or increasing public familiarity with program requirements, cannot be determined from aggregate data and warrant further investigation.

The annual demographic profile consistently skews toward midlife adults (35–49 years) and women, mirroring trends in the broader “wellness” psychedelic landscape (18). The concentration of participants in these age brackets, combined with the significant “service tourism” component where 32.6% of clients originated from outside Oregon, suggests that financial resources may be a primary driver of access. The estimated high cost of regulated services likely favors more mature clients with higher disposable income, while younger demographics may continue to rely on lower-cost naturalistic use. Furthermore, the substantial representation of LGBTQ+ clients (reaching 32.4% in Q1) suggests the program provides a critical pathway for a population that historically experience higher rates of mental health distress (19). This engagement may reflect a specific openness within the LGBTQ+ community toward alternative wellness modalities and “novel experiences” within a regulated framework (20). While aggregate data preclude a direct correlation between sexual orientation and specific motivations for use, this engagement may be influenced by Oregon’s regulatory requirements for facilitator training, which mandate education on LGBTQIA2S+ cultural resilience and affirming care (OAR 333-333-3050) (21). Whether this reflects a specific seeking of identity affirmation or a response to a culturally competent service model remains a vital question for longitudinal research.

Racial and ethnic diversity remains a significant challenge for the program, likely exacerbated by the high cost of regulated services. White and European-descended clients accounted for 84.1% to 91.5% of the reporting population across all quarters, significantly exceeding Oregon’s ‘White alone’ census benchmark of 61.6%. This demographic skew mirrors the persistent lack of diversity in formal psilocybin clinical trials, where White participants have been reported to represent up to 87.2% of study cohorts (22). The parallel between Oregon’s regulated ‘wellness’ model and the clinical research environment suggests that structural barriers, such as the high out-of-pocket cost of services, may be more significant drivers of exclusion than traditional medical inclusion or exclusion criteria. While African American participation (approx. 2.2%–2.4%) lags national demographics, it aligns closely with Oregon’s specific population (approx. 2%). Conversely, Hispanic/Latino representation in the study (approx. 6%–9% overall; 5.6% by Q4) appears significantly lower than both the state and national census benchmarks of 18.7% and 20%, respectively (23). Interpretations of this disparity must consider the 32.6% service tourism rate; the inclusion of out-of-state and international clients introduces a demographic variable distinct from the state census. Our finding that approximately one-third of clients are travelers suggests that a ‘wealth gap’ in access persists, where the financial resources required for participation, including service fees, airfare, and lodging, disproportionately favor more affluent demographics. To address these barriers, the Oregon framework includes specific equity mandates, such as requiring all facilitators to complete 12 hours of ‘Cultural Equity’ and ‘Social Justice’ training (OAR 333-333-3050) (21). Additionally, the state has implemented a tiered licensing fee structure for veterans and low-income providers to increase provider diversity and reduce costs (OAR 333-333-4060) (24). While these policy tools are foundational, our findings underscore that training and fee adjustments alone have not yet resulted in a participant base that mirrors state or national diversity, highlighting the need for continued focus on affordability and community-specific outreach.

The reported reasons for use highlight a convergence of therapeutic and non-clinical motivations. While “general wellness” and “change of perspective” were the most frequent drivers, aligning with naturalistic use studies (25, 26), approximately 20% to 25% of clients cited anxiety or depression. This aligns with clinical literature demonstrating psilocybin’s efficacy for these conditions (2–4, 18, 27–30), yet it occurs here within a service-oriented framework that does not require a medical diagnosis (5). This suggests that many clients may be functionally seeking therapeutic outcomes through a non-medical pathway, particularly given the high prevalence of unmet mental health needs (16, 17). Emerging interests in creativity and spirituality are also supported by prior research on psilocybin’s transformational effects (31, 32).

The substantial representation of out-of-state and international clients (nearly one-third of the annual total) highlights the national and global reach of Oregon’s first-in-the-nation regulatory framework. The peak in domestic tourism during Q3 suggests a growing national awareness of the program, even as overall quarterly session volumes stabilized. This high rate of “psychedelic tourism” further complicates the assessment of health equity when using state-level census data as a benchmark, as the resources required for interstate or international travel likely correlate with higher socioeconomic status. These findings emphasize that the OPS program serves as a critical access point not only for Oregonians but for a broader geographic population seeking supervised psilocybin services in a regulated environment.

Psilocybin-assisted therapy has shown rapid, lasting benefits for depression, anxiety, substance use disorders, and end-of-life distress in multiple studies (2, 33–35). Oregon, facing high rates of mental illness and unmet needs, created the first statewide system for supervised psilocybin services with strict safety standards (1, 16, 17, 36). The OPS model demonstrates that statewide supervised psilocybin services can be implemented safely, with transparent data supporting ongoing policy development and public confidence. Despite not being a medical program, many clients seek help for mental health concerns, suggesting future collaboration with healthcare systems may be needed to ensure continuity of care (34). As data collection expands, researchers will be able to assess equity and identify barriers to access, especially for marginalized groups. However, OPS lacks validated outcome measures, highlighting the need for future longitudinal research on symptom and functioning changes. The continued utilization of group sessions (**Table 1**) also raises questions about their cost-effectiveness, social benefits, and safety compared to individual administration, warranting further study.

### 4.1 Limitations

These findings are limited by the nature of aggregate, cross-sectional, and de-identified data, which restrict the ability to track individual outcomes, assess long-term safety, or evaluate the clinical trajectory of clients reporting mental health conditions. Because OPS collects no validated outcome measures, these data cannot assess whether services achieve therapeutic goals, and we do not interpret utilization patterns as evidence of clinical effectiveness. While we present a summary of racial and ethnic demographics, the exclusion of opt-out clients and privacy-mandated suppression of data for small cell sizes (counts <10) precludes a complete assessment of equity. Because participation in data collection is voluntary, nonrespondents may differ in key demographics, limiting equity inferences. Consequently, the categories presented likely underrepresent specific racial or ethnic subgroups with lower utilization rates.

Furthermore, significant data gaps exist; for instance, gender identity and sexual orientation data were missing for a substantial portion of the population, ranging from approximately 3% to 19% depending on the quarter. This level of missingness, necessitates caution in generalizing these demographic characteristics to the entire OPS client base. Additionally, the OPS reporting structure introduces several challenges:

- Voluntary client participation in data collection may result in selection bias.
- Motivations and demographic details are self-reported and subject to social desirability or misunderstanding.
- The release of only aggregate data prevents correlation analyses, multivariable modeling, longitudinal follow-up, or intersectional demographic analyses (such as age by race by gender). The lack of distinction between residents and non-residents in certain demographic domains makes direct comparisons to state-level census data preliminary rather than definitive.
- Privacy protections and the lack of validated clinical outcome measures mean the OPS program can currently track only acute adverse events, not long term impact on symptoms or functioning.
- The lack of unique longitudinal identifiers in the statewide aggregate data prevents an assessment of repeated service use or the prevalence of multi-dose protocols. This means that the total annual ‘clients served’ may include returning individuals counted across different quarters, potentially influencing the reported demographic proportions and motivations. Future research utilizing individual-level data will be required to distinguish between unique and returning participants to better understand the clinical and social impact of the program.
- Finally, while safety events remained rare, the low volume of events may also reflect limitations in the quality or completeness of reporting within a non-medical framework. Inclusion of licensees without medical or mental health provider credentials represents an important deviation from the personnel usually assigned to determine causality of adverse events in clinical research and practice. Consequently, the absence of standardized clinical assessment may lead to the underreporting or misclassification of psychological and physiological distress as non-adverse phenomena.

Despite these inherent constraints, the OPS dataset currently offers the most comprehensive real-world information on regulated psilocybin services available. While future updates, including data on veteran status, will improve interpretability, OPS currently offers the most comprehensive real-world information on psilocybin services available, despite these inherent constraints (32, 34, 35).

## 5 Conclusions

Oregon’s psilocybin services program stands as a pioneering statewide model for regulated psychedelic services that seeks to promote safety, informed consent, privacy, and equity. Full-year statewide data from 2025 reveal a stabilized pattern of utilization following initial growth, characterized by consistently low rates of acute adverse behavioral and medical events. While the program has successfully reached notable proportions of LGBTQ+ and gender-diverse individuals, racial and ethnic diversity lags significantly behind state demographics, and the high rate of service tourism (32.6%) suggests that geographic and socioeconomic barriers to access persist.

The findings suggest that the OPS program serves a dual role: it functions as a regulated wellness modality for personal growth while simultaneously acting as a functional alternative for individuals seeking to address mental health distress outside of traditional medical pathways. These findings provide foundational evidence for policymakers, clinicians, and researchers in other jurisdictions considering similar regulatory frameworks. However, addressing the “wealth gap” in access, refining the precision of safety reporting, and implementing longitudinal research on clinical outcomes remain essential to fully understand the long-term public health implications of supervised psilocybin services.

## Data Availability

Publicly available datasets were analyzed in this study. This data can be found here: Oregon Psilocybin Services Data Dashboard Archive: https://www.oregon.gov/oha/PH/PREVENTIONWELLNESS/Pages/Psilocybin-Data-Archive.aspx.
