# Supplementary material for: Inaugural year of regulated psilocybin services in Oregon: safety, motivations, and utilization
Source: Front Psychiatry. 2026 May 13;17:1777387. doi: 10.3389/fpsyt.2026.1777387 (PMC13224104; doi:10.3389/fpsyt.2026.1777387)
Supplement: Supplementary file 1 [file Table1.docx]

**Supplemental Data 1**

**Inaugural Year of Regulated Psilocybin Services in Oregon: Safety, Motivations, and Utilization**

**Feliciano Yu, MD ^1, 2^, Joe Tafur, MD ^2^, Francisco Moreno, MD ^3^, Stephen Dahmer, MD ^2^**

^1^ University of Arkansas for Medical Sciences, Department of Pediatrics, Little Rock, Arkansas

^2^ University of Arizona, Andrew Weil Center for Integrative Medicine, Tucson, Arizona

^3^ University of Arizona, College of Medicine, Tucson, Arizona

**Table S1** Client-reported motivations and reasons for seeking Oregon Psilocybin Services (OPS), by quarter (2025 Q1–Q4).

| **Motivation** | **Q1 (n)** | **Q2 (n)** | **Q3 (n)** | **Q4 (n)** | **Annual Total (n)** | **Annual %** |
| --- | --- | --- | --- | --- | --- | --- |
| **Wellness** | 488 | 447 | 469 | 412 | **1,816** | **30.6%** |
| **Perspective** | 397 | 459 | 394 | 392 | **1,642** | **27.7%** |
| **Consciousness** | 404 | 427 | 388 | 383 | **1,602** | **27.0%** |
| **Anxiety** | 375 | 404 | 328 | 306 | **1,413** | **23.8%** |
| **Depression** | 358 | 366 | 286 | 294 | **1,304** | **22.0%** |
| **Creativity** | 240 | 213 | 210 | 225 | **888** | **15.0%** |
| **Spirituality** | 223 | 250 | 206 | 179 | **858** | **14.5%** |
| **PTSD** | 213 | 238 | 175 | 149 | **775** | **13.1%** |
| **Exhaustion** | 147 | 183 | 158 | 171 | **659** | **11.1%** |
| **Other Trauma** | 131 | 169 | 137 | 147 | **584** | **9.8%** |

Note - Reasons are not mutually exclusive; clients could select multiple categories; therefore, percentages may sum to >100%. Percentages are calculated as category count divided by clients served in the quarter, excluding suppressed values (coded as −99).

**Table S2.** Analysis of Out-of-State and International Service Tourism (2025)

| **Quarter** | **Clients Served** | **Other Inside US (n)** | **Outside US (n)** | **Total Tourism (%)** |
| --- | --- | --- | --- | --- |
| 2025 Q1 | 1,509 | 369 | Suppressed* | 24.5% |
| 2025 Q2 | 1,758 | 448 | 140 | 33.4% |
| 2025 Q3 | 1,310 | 483 | 21 | 38.5% |
| 2025 Q4 | 1,358 | 453 | 22 | 35.0% |
| **Annual Total** | **5,935** | **1,753** | **183** | **32.6%** |

*Q1 International data suppressed due to small cell size (<10).
